# Prefrontal activation during dual-task seated stepping and walking performed by subacute stroke patients with hemiplegia

**DOI:** 10.3389/fnins.2023.1169744

**Published:** 2023-05-05

**Authors:** Shinnosuke Nosaka, Ken Imada, Kazuya Saita, Hitoshi Okamura

**Affiliations:** ^1^Graduate School of Biomedical and Health Sciences, Hiroshima University, Hiroshima, Japan; ^2^Kinkai Rehabilitation Hospital, Yonago, Japan

**Keywords:** dual task, seated stepping, stroke, subacute, near-infrared spectroscopy, prefrontal cortex, gait

## Abstract

**Objectives:**

This study examined prefrontal cortex (PFC) activation during dual-task seated stepping and walking performed by subacute stroke patients with hemiplegia and evaluated the relationship between PFC activation, frontal lobe functions, and dual-task interference.

**Methods:**

Patients with functional ambulation category (FAC) scores ≤ 2 comprised the seated stepping task group. Those with FAC scores > 2 comprised the walking task group. There were 11 patients in the seated stepping task group (mean age, 65.3±12.2 years; age range, 55-73.5 years; 7 male and 4 female patients; time since stroke onset, 45.7±9.9 days) and 11 patients in the walking task group (mean age, 65.6±15.2 years; age range, 49.5-74.5 years; 7 male and 4 female patients; time since stroke onset, 57.5±18.3 days). Both groups completed the Frontal Assessment Battery (FAB). The seated stepping task group performed the following three tasks: cognitive task (CT), normal seated stepping (NSS), and dual-task seated stepping (DTSS). The walking task group completed the following tasks: CT, normal walking (NW), and dual-task walking (DTW). The CT was a letter fluency task; this letter fluency task was simultaneously performed during seated stepping (DTSS) and walking (DTW). Changes in the oxygenated hemoglobin (O_2_Hb) concentration and deoxygenated hemoglobin concentration during the tasks were measured using near-infrared spectroscopy (Pocket NIRS HM; Dynasense Inc., Japan). The number of steps, walking speed, and percentage of correct responses to the CT were recorded.

**Results:**

The results showed that DTSS activated the PFC significantly more than performing a single task and that NSS was associated with a significantly higher difference in the hemoglobin concentration when compared to that associated with the CT, which was a single task. In the walking task group, PFC activation was significantly higher during DTW, NW, and CT (in that order), and O_2_Hb concentrations were significantly higher in the contralesional hemisphere than in the ipsilesional hemisphere during all tasks. Associations between PFC activation, FAB scores, and dual-task interference in the seated task group indicated significant positive correlations between FAB scores and cognitive performance with dual-task interference.

**Conclusion:**

DTSS may be an effective means of activating the PFC of patients with difficulty walking.

## 1. Introduction

When performing dual tasks (motor and cognitive tasks (CTs) performed simultaneously), stroke patients exhibit significantly poorer performance than healthy older adults (Plummer et al., 2013). It has been reported that dual tasks activate the prefrontal cortex (PFC; Yogev-Seligmann et al., 2008). Dual-task training for stroke patients has been shown to effectively improve dual-task gait function and decrease the incidence of falls (Pang et al., 2018). Within the PFC, the frontal pole is involved in cognitive processes that lead to voluntary behavior (Zysset et al., 2002; Haggard, 2008). The frontal pole is also preferentially activated when learning new motor tasks (Ishikuro et al., 2014) and has an important role in rehabilitation.

Functional near-infrared spectroscopy (fNIRS) has been used in several studies of PFC activity, especially during walking, because it offers a greater degree of freedom when performing measurements and allows for real-time measurements (Pelicioni et al., 2019; Lim et al., 2022). Furthermore, among fNIRS instruments, wireless devices allow measurements not only while walking on a treadmill but also while walking at comfortable speeds in natural environments (Nosaka et al., 2022). The fNIRS studies that have evaluated changes in the oxygenated hemoglobin (O_2_Hb) concentrations of stroke patients reported greater changes in the PFC during dual tasks than during the performance of a single task (Al-Yahya et al., 2016; Hawkins et al., 2018; Liu et al., 2018). Furthermore, patients with poor motor function rely more on the cortical control of gait (Miyai et al., 2002), and PFC activation in stroke patients is correlated with physical performance (Mori et al., 2018).

Studies involving subacute stroke patients have shown an upper limit of PFC oxygenation during walking alone (Hermand et al., 2019); however, few studies of subacute stroke patients have been performed. Many subacute stroke patients undergoing the process of functional recovery have difficulty walking. Seated stepping is easy to perform, even for patients with difficulty walking, because of the low risk of falling, and dual-task seated stepping (DTSS) activates the PFC (Ohsugi et al., 2013). A randomized controlled trial that introduced stepping as an exercise showed that DTSS effectively improved gait performance compared to stepping alone (Yamada et al., 2012). However, these studies involved healthy older subjects living in the community. There are no reports of PFC activation during seated stepping tasks performed by stroke patients.

The main objective of this study was to examine PFC activation during DTSS and dual-task walking (DTW) performed by subacute stroke patients with hemiplegia. The secondary objective was to evaluate the association between PFC activation, frontal lobe functions, and dual-task interference. It was hypothesized that patients with poorer motor function would experience increased PFC activation during DTSS as well as during DTW. It was also hypothesized that there would be an association between PFC activation, frontal lobe functions, and dual-task interference. If the PFC is activated by seated stepping while performing a CT as well as by walking while performing a CT, then exercises for the rehabilitation of stroke patients who have difficulty walking could be optimized.

## 2. Materials and methods

### 2.1. Participants

Subacute stroke patients who met the following selection criteria were included in the study: aged 20 years or older; consented to participate in the study; experienced a unilateral stroke with first hemiparesis; experienced stroke onset within 2 weeks to 3 months of the study (Hermand et al., 2019); medically stable; able to perform seated stepping; able to understand the task; and right-hand-dominant. Exclusion criteria were multiple stroke lesions, orthopedic disease, pre-existing central nervous system disease, aphasia, and cognitive impairments (Mini-Mental State Examination score < 21; Hawkins et al., 2018). Subjects with Functional Ambulation Categories (FAC) scores ≤ 2 comprised the seated stepping task group; those with FAC scores > 2 comprised the walking task group (Holden et al., 1984).

An estimation of the sample size using G Power (a power analysis software) indicated that 11 subjects per group were required (significance level = 0.05; statistical power = 0.95; mean and standard deviation [SD] for group 1 = 11.4 and 1.31; mean and SD for group 2 = 9.0 and 1.55; Ishikuro et al., 2014; Ota et al., 2020). It has also been reported that the criteria quality is higher with a sample size of 10 or more per group (Pelicioni et al., 2019). Therefore, a total of 22 (11 in the seated stepping task group and 11 in the walking task group) subjects were included.

### 2.2. Measurements

#### 2.2.1. Clinical data

Demographic data (such as age, sex, height, and weight) and clinical characteristics (such as stroke subtype, lesion hemisphere, lesion depth, number of days since stroke onset, and FAC score as a measure of walking ability) were obtained from the electronic medical records (Holden et al., 1984).

#### 2.2.2. Prefrontal function

Frontal lobe functions were assessed by the Frontal Assessment Battery (FAB), which was developed by Dubois et al. (2000) as a brief screening tool for various components of frontal lobe function and consists of the following six subtests: similarities, word fluency, motor series, conflicting instructions, go/no go tasks, and prehension behavior. Each subtest is scored using a scale ranging from 0 to 3, with the overall score ranging from 0 to 18; higher scores indicate better frontal lobe function. The reliability and validity of the Japanese version of this test for identifying frontal lobe dysfunction have been confirmed (Nakaaki et al., 2007).

#### 2.2.3. Single and dual tasks

The seated stepping task group performed the following three tasks: CT, normal seated stepping (NSS), and DTSS. The CT was a letter fluency task that facilitated word recall using the first letter of the word (e.g., those beginning with “a”) while seated in a chair. NSS was performed while seated in a standard dining room chair (41 cm high); only the nonparalyzed lower limbs were moved up and down at a comfortable rate. The minimum lifting height for stepping was the height between the plantar surface of the foot and the ground. During DTSS, a letter fluency task (CT) and seated stepping were simultaneously performed. The walking task group performed the following three tasks: CT, normal walking (NW), and DTW. During NW, the subject walked for 20 meters along a path at a comfortable walking speed, changed direction at the end of the path, and continued walking until the end of the walking period. DTW comprised the simultaneous performance of NW and CT. During DTW, a physical therapist remained 80 cm behind the subject to help prevent falls. The duration of each task was 30 s. Both groups performed three sets of each of the three tasks in random order.

### 2.3. Task performance

During the seated stepping task, the number of steps performed within 30 s was counted. During the walking task, the distance walked by the subject during the 30-s walking period was measured, and the walking speed (m/s) was calculated based on the distance. During the letter fluency task, responses were recorded with a voice recorder, and the percentage of correct responses was calculated as follows (Pang et al., 2018; Equation 1):


(1) $$\begin{array}{l} Percentageofcorrectanswers\;\left(\%\right)=\\ \;\;\;\;\;\;\;\;\;\;\;\;\;\;\;\;\;\;\;\;\;\;\;\;\;\;\;\frac{Numberofcorrectanswers\;}{Time\;\left(seconds\right)}\times 100 \end{array}$$

The effect of the dual task was considered dual-task interference and calculated as follows (Rochester et al., 2014; Equation 2):


(2) $$\begin{array}{l} interference_{DT}=performance_{DT}\\ \;\;\;\;\;\;\;\;\;\;\;-performance_{ST\;\left(DT:dualtask,\;\;ST:standardtask\right)} \end{array}$$

### 2.4. Near-infrared spectroscopy measurements and analysis

During this study, we used the Pocket NIRS HM (Dynasense Inc., Hamamatsu, Japan), which is a functional near-infrared spectroscopy (fNIRS) instrument that can noninvasively measure cerebral blood flow changes associated with neural activity. The Pocket NIRS HM measures changes in relative O_2_Hb, deoxygenated hemoglobin (HHb), and total hemoglobin (Hb) concentrations. Measurements were obtained at the prefrontal 1 and prefrontal 2 sites of the international 10/20 system (equivalent to Brodmann area 10 at the bilateral frontal poles) using a dedicated head-mounted (two-channel) continuous-wave method with light-emitting diodes at three wavelengths (735, 810, and 850 nm). Changes in hemoglobin concentration were determined using the modified BeerLambert law. The distance between the light source and the detector was 3 cm. Data were transferred to a smartphone using Bluetooth wireless communication techniques; the sampling rate was 10 Hz (Mirelman et al., 2014, 2017; Nosaka et al., 2022). The blood pressure and heart rate were measured before and after the start of each task; the CT, NSS, and DTSS were performed in the seated position, whereas NW and DTW were performed while in the standing position. These positions may have affected the near-infrared spectroscopy (NIRS) measurements. The measured raw data included artifacts such as respiration, heart rate, body movement, and jaw movement. Data output from the Pocket NIRS HM (in csv format files) were exported to MATLAB R2019b (MathWorks Inc., Natick, MA, United States). These artifacts were removed by a multiresolution analysis using the Wavelet Toolbox (Nosaka et al., 2022). During the multiresolution analysis, the data were decomposed down to level 4 using the sym4 wavelet to produce approximations and differences. A block design was used for the experimental design. Baseline measurements were obtained during the first 10 s of the 20-s resting period before task initiation and the last 10 s of the 20-s resting period after task completion (Doi et al., 2013). To assess relative task-related O_2_Hb and HHb concentrations, the average concentration at each baseline value was subtracted from the concentration observed during task performance (Mirelman et al., 2017), and the values of the summation averages from three repetitions of each task were calculated (Doi et al., 2013; Mori et al., 2018). From these data, hemoglobin differences (differences between the O_2_Hb values and HHb values) and O_2_Hb concentration differences in the ipsilesional and contralesional hemispheres were extracted (Holtzer et al., 2011; Lu et al., 2015; Hermand et al., 2019, 2020).

### 2.5. Statistical analysis

Normally distributed data of the continuous variables collected are presented as the mean (± SD); those with non-normal distribution are presented as the median (interquartile range [IQR] comprising the 25th–75th percentiles). Comparisons of demographic data, clinical information, FAB scores, correct response rates, and dual-task interference between groups were performed. Student’s *t*-test or Wilcoxon’s rank sum test was performed depending on whether the data were normally or non-normally distributed. Paired *t*-tests were performed to compare the number of steps during NSS and DTSS within groups, correct response rates during the CT and DTSS, correct response rates during the CT and DTW, and blood pressure and heart rate before and after the start of the tasks, and walking speeds during NW and DTW were analyzed using Wilcoxon’s signed rank test. Differences in O_2_Hb concentrations associated with tasks and the hemoglobin and O_2_Hb concentrations in the ipsilesional and contralesional hemispheres were analyzed using mixed models. Tasks and hemispheres were used as fixed effects; participants were used as random effects. Correlations between PFC activation and dual-task interference with frontal lobe function and task performance were analyzed using Pearson’s correlation coefficient or Spearman’s rank correlation coefficient, depending on the distribution. All statistical analyses were performed using JMP® Pro 16 (SAS Institute Inc., Cary, NC, United States); *p* < 0.05 was considered significant.

## 3. Results

### 3.1. Clinical data

Twenty-two patients who met the selection criteria and provided consent to participate in the study were included in the analysis. The only significant difference between the seated stepping task and walking task groups was observed for the FAC score (Table 1).

**Table 1:** Comparison of clinical data of the seated stepping task and walking task groups.

	Seated stepping task group (*n* = 11)	Walking task group (*n* = 11)	*p-*value
Age (years)	65.3 ± 12.2	65.6 ± 15.2	0.974
Sex (male/female)	7/4	7/4	1
Height (cm)	161.6 ± 9.3	162.7 ± 10.6	0.813
Weight (kg)	61.9 ± 10.8	56.9 ± 10.6	0.283
FAC score (1/2/3/4/5)	3/8/0/0/0	0/0/2/7/2	<0.001
MMSE (maximum, 30)	26 (25.5–28)	28 (28–29.5)	0.096
Subtype (ischemic/hemorrhagic)	7/4	4/7	0.201
Lesion hemisphere (left/right)	3/8	4/7	0.647
Lesion depth (cortical/subcortical/mixed)	1/6/4	0/9/2	0.322
Time from stroke onset (days)	45.7 ± 9.9	57.5 ± 18.3	0.077

Mean ± standard deviation or median [interquartile range (25th–75th quartiles)]. FAC, Functional Ambulation Categories; MMSE, Mini-Mental State Examination.

### 3.2. Prefrontal functions and task performances

A comparison of the two groups showed no significant differences in the FAB scores, correct response rates, and perception of dual-task interference (Table 2). In the seated stepping task group, the mean numbers of steps during NSS and DTSS were 46.0 ± 15.9 and 42.1 ± 13.5, respectively; this difference was not significant (*p* = 0.406). The median number of steps performed with dual-task interference was −3.7 (IQR, −4.8 to 3.5). At the beginning of the study, the mean systolic blood pressure was 124.6 ± 12.3 mmHg, the mean diastolic blood pressure was 77.8 ± 10.0 mmHg, and the mean heart rate was 71.7 ± 11.5 beats/min. At the end of the study, the mean systolic blood pressure was 123.1 ± 13.7 mmHg, the mean diastolic blood pressure was 80.8 ± 8.3 mmHg, and the mean heart rate was 72.3 ± 13.6 beats/min. Systolic blood pressure and heart rate at the beginning of the study were not significantly different from those at the end of the study (*p* = 0.658 and *p* = 0.599, respectively); however, diastolic blood pressures at the beginning and the end of the study were significantly different (*p* = 0.047).

**Table 2:** Comparison of FAB scores, correct response rates, and perception of dual-task interference of the seated stepping task and walking task groups.

	Seated stepping task group (*n* = 11)	Walking task group (*n* = 11)	*p-*value
FAB (maximum, 18)	17 (15–17)	15 (13.5–17)	0.499
**Correct response rate (%)**			
CTDTSS/DTW	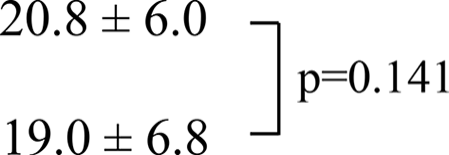	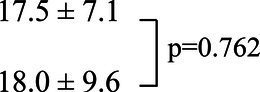	0.2490.779
Perceived dual-task interference (%)	˗1.8 ± 3.8	0.5 ± 5.4	0.259

Mean ± standard deviation or median (25th–75th quartiles). Correct response rates were those of the letter fluency task. CT, cognitive task; DTSS, dual-task seated stepping; DTW, dual-task walking; FAB, Frontal Assessment Battery.

In the walking task group, the median walking speeds of 0.82 m/s (IQR, 0.76–0.85 m/s) for NW and 0.7 m/s (IQR, 0.66–0.73 m/s) for DTW were significantly different (*p* = 0.001); the median walking speed with dual-task interference was −0.11 m/s (IQR, −0.13 to −0.08 m/s). During the study, the mean systolic and diastolic blood pressures changed from 134.0 ± 16.6 to 128.5 ± 13.8 mmHg (*p* = 0.195) and 78.7 ± 7.2 to 79.8 ± 7.5 mmHg (*p* = 0.539), respectively, and the mean heart rate changed from 72.9 ± 14.5 to 70.2 ± 15.2 beats/min (*p* = 0.347).

### 3.3. Near-infrared spectroscopy data

**Figure 1:**
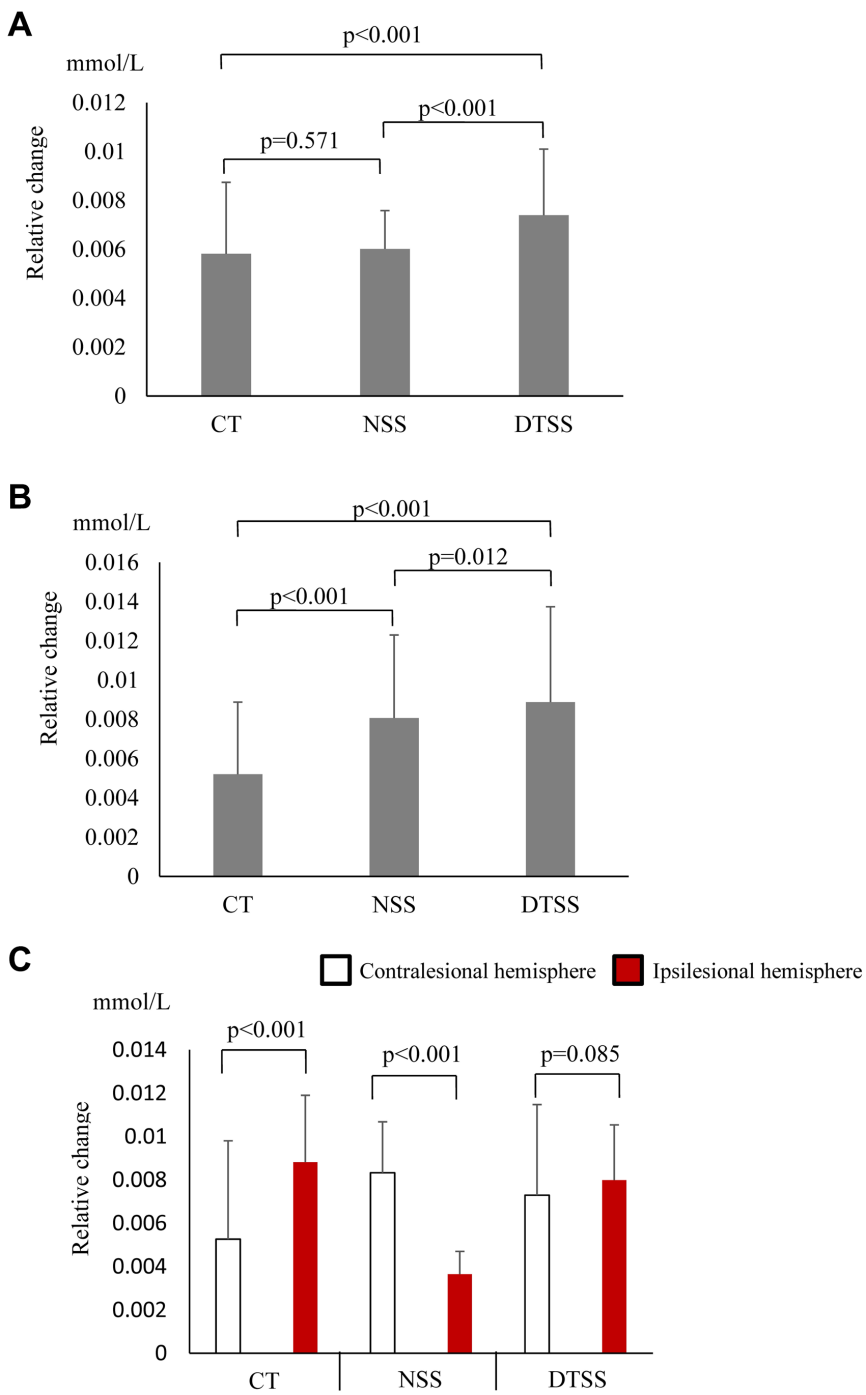
**(A)** Oxygenated hemoglobin (O_2_Hb) concentrations (mean ± standard deviation) observed with tasks performed by the seated stepping task group. CT, cognitive task; DTSS, dual-task seated stepping; NSS, normal seated stepping. **(B)** Difference in hemoglobin concentrations observed with tasks performed by the seated stepping task group (mean ± standard deviation). CT, cognitive task; DTSS, dual-task seated stepping; NSS, normal seated stepping. **(C)** Inter-task and inter-hemispheric oxygenated hemoglobin (O_2_Hb) concentrations (mean ± standard deviation) of the seated stepping task group. CT, cognitive task; DTSS, dual-task seated stepping; NSS, normal seated stepping.

In the seated stepping task group, O_2_Hb concentrations showed a significant main effect of the task (*p* < 0.001), and O_2_Hb concentrations during DTSS were significantly higher than those during the CT and NSS (Figure 1A). The difference in hemoglobin concentrations also showed a significant main effect of the task (*p* < 0.001), with significantly higher values observed during NSS and DTSS compared to those observed during the CT (Figure 1B). There was no main effect of the hemisphere on the differences in O_2_Hb concentrations in the ipsilesional and contralesional hemispheres (*p* = 0.957; Figure 1C).

**Figure 2:**
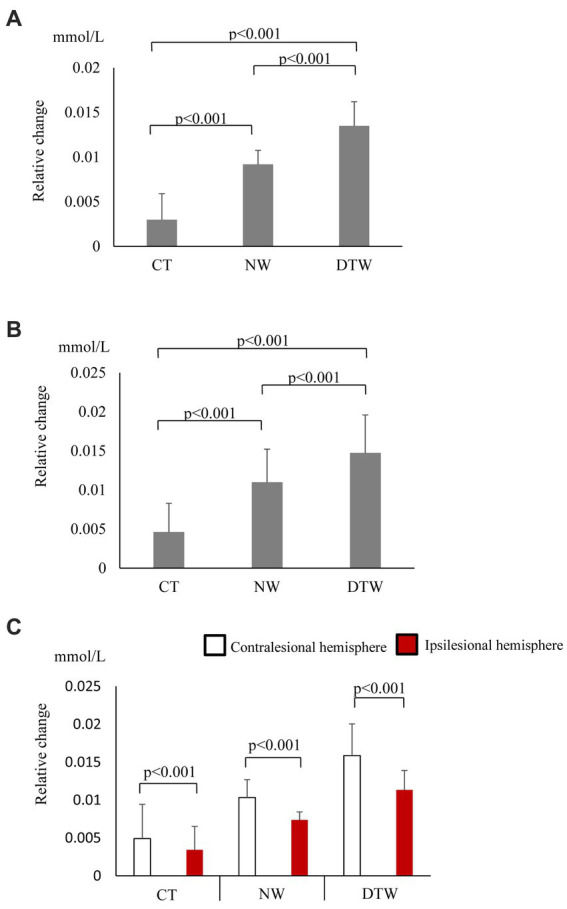
**(A)** Oxygenated hemoglobin (O_2_Hb) concentrations (mean ± standard deviation) observed with tasks performed by the walking task group. CT, cognitive task; DTW, dual-task walking; NW, normal walking. **(B)** Hemoglobin concentration differences observed with tasks performed by the walking task group (mean ± standard deviation). CT, cognitive task; DTW, dual-task walking; NW, normal walking. **(C)** Inter-task and inter-hemispheric oxygenated hemoglobin (O_2_Hb) concentrations of the walking task group (mean ± standard deviation). CT, cognitive task; DTW, dual-task walking; NW, normal walking.

**Figure 3:**
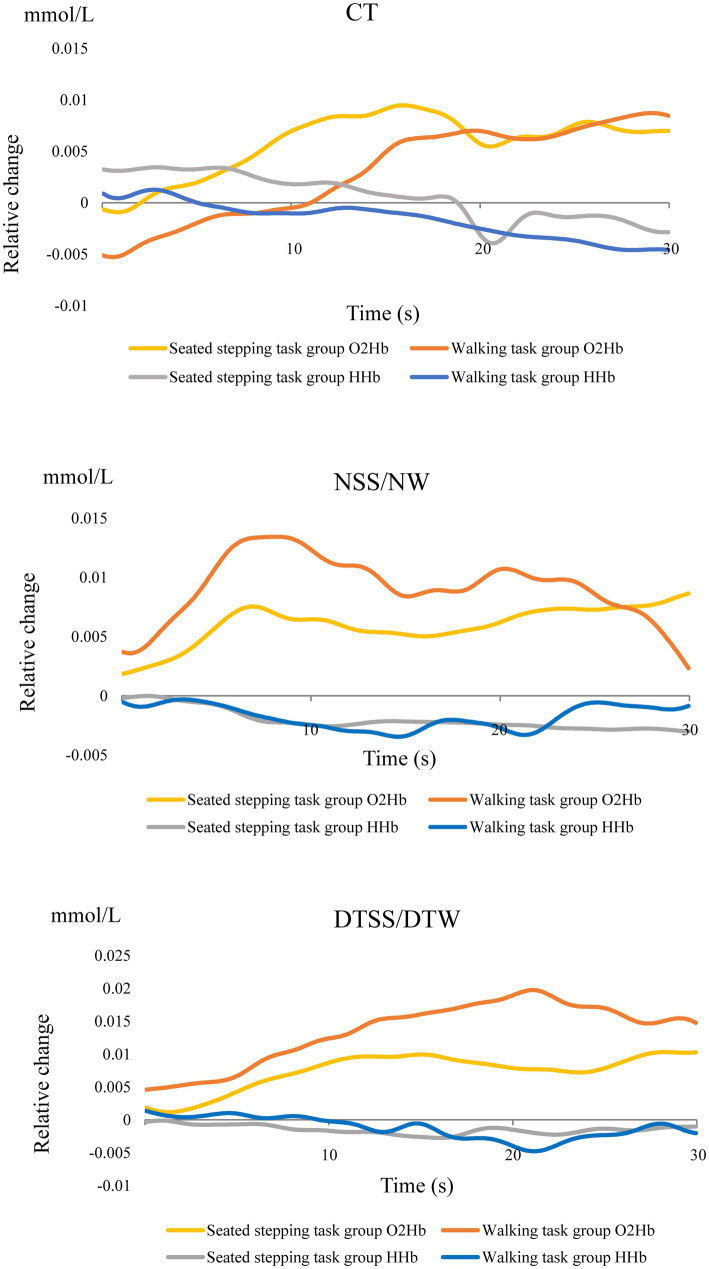
Relative changes in O_2_Hb and HHb concentrations during the task.

In the walking task group, O_2_Hb concentrations showed a significant main effect of the task (*p* < 0.001); these increased significantly during walking and were most significantly higher during DTW (Figure 2A). The difference in hemoglobin concentrations showed a significant main effect of the task (*p* < 0.001) and, similar to the O_2_Hb concentrations, increased significantly during walking and was most significantly higher during DTW (Figure 2B). The difference in O_2_Hb concentrations between the ipsilesional and contralesional hemispheres showed a significant main effect of the hemisphere (*p* < 0.001), with the contralesional hemisphere showing significantly higher values during all tasks (Figure 2C). Relative changes in O_2_Hb and HHb concentrations during the task are shown in Figure 3.

### 3.4. Correlations among prefrontal cortex activation, prefrontal function, and dual-task interference

**Figure 4:**
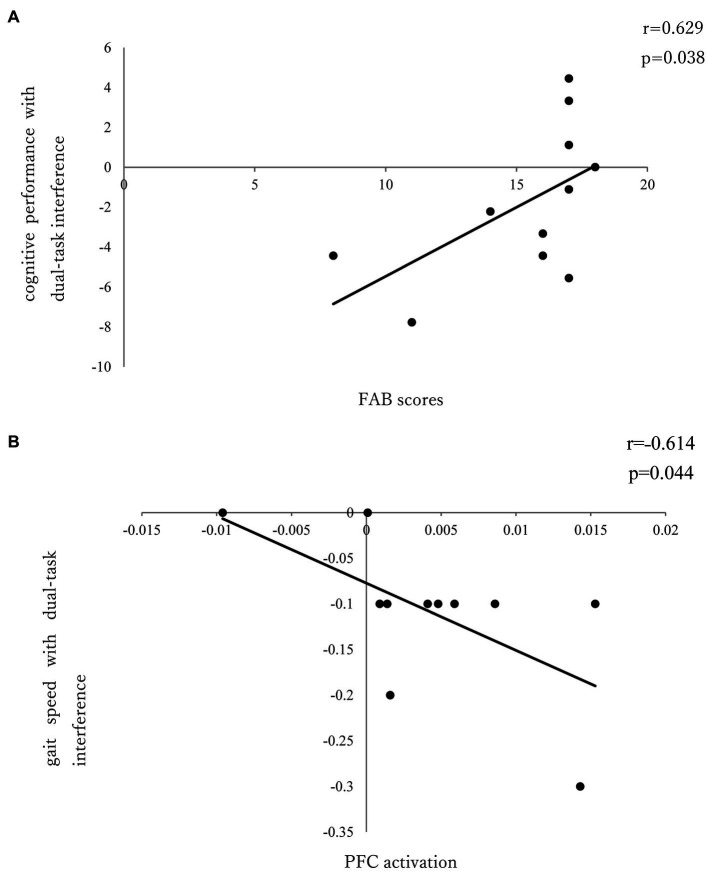
**(A)** Correlation between FAB and cognitive performance of dual-task interference. **(B)** Correlation between PFC activation and gait speed of dual-task interference.

There was no significant correlation between dual-task interference with PFC activation and task performance in the seated stepping task group. No significant correlation was found between PFC activation and FAB scores; however, a significant positive correlation was observed between FAB scores and cognitive performance with dual-task interference (r = 0.629; *p* = 0.038; Figure 4A).

In the walking task group, there was a significant negative correlation (r = ˗0.614; *p* = 0.044) between PFC activation and gait speed with dual-task interference (Figure 4B); however, there was no significant correlation between PFC activation and FAB scores or between FAB scores and dual-task interference.

## 4. Discussion

These results are novel; they showed that compared to seated stepping alone, DTSS significantly activated the PFC among subacute stroke patients with hemiplegia. To our knowledge, this is the first study to examine PFC activation during seated stepping among subacute stroke patients. Interestingly, in terms of differences in hemoglobin concentrations, those during NSS were significantly higher than those during the CT. In the seated stepping task group, significant positive correlations between FAB and cognitive performance with dual-task interference were observed.

Ohsugi et al. (2013) examined PFC activation during seated stepping by single-task (computation or seated stepping) and dual-task (both single-tasks performed simultaneously) in healthy young and older subjects and found no PFC activation by seated stepping alone in both groups, but PFC was activated by the computational task and the dual task. Activation by the dual task was more pronounced in healthy older adults than in younger participants. These results indicate that the higher load required by the dual task induces PFC activity in older adults, whose cognitive and motor functions are lower than those of younger subjects. Therefore, the present study hypothesized that patients with low motor function and difficulty walking would show PFC activation during dual-task seated stepping, and the results showed that dual-task seated stepping significantly activated PFC compared to that by single-task stepping. In this study, those with cognitive impairment were excluded, and we believe that the results indicate that in stroke patients with relatively preserved cognitive function, the upper limit of PFC oxygenation was not reached in single tasks such as CT and NSS, as in healthy older subjects. However, NSS resulted in a significantly higher hemoglobin concentration difference than CT did, a result different from that reported by Ohsugi et al. (2013). Mori et al. (2018) examined PFC activation during double-task gait in stroke hemiplegic patients and reported that PFC activation correlated with physical performance. In the walking task group in this study, PFC activation was highest during DTW; however, the PFC was more activated during NW than during CT. In the seated stepping task group with low motor function and difficulty walking, the seated stepping task involves similar physical demands as walking, which may be why the NSS resulted in a significantly higher hemoglobin concentration difference than the CT did. Moreover, significant positive correlations between FAB and cognitive performance with dual-task interference were observed. Nosaka et al. (2022) examined PFC activation during DTW by grouping community-dwelling older individuals using the cutoff value of the Japanese version of the Montreal Cognitive Assessment and found that the group with scores ≥ 26 and higher FAB scores showed significantly higher O_2_Hb concentrations during DTW compared to those during NW, whereas the group with scores < 26 and lower FAB scores showed no increase in O_2_Hb concentrations during DTW compared to those during NW. These findings suggest that higher frontal lobe function, as indicated by FAB, leaves more unused brain resources for dual-tasking, which may be a factor in the significant correlation between FAB and cognitive performance on dual-task interference. Furthermore, it was suggested that unused brain resources may be prioritized for the cognitive task. In the walking task group, PFC activation was significantly higher during DTW, NW, and CT (in that order), and significant negative correlations were found between PFC activation and gait speed in dual-task interference. A study that examined PFC activation during DTW performed by subacute stroke patients (Hermand et al., 2019) reported that PFC oxygenation showed an upper limit during walking alone, and it did not increase further when performing dual tasks. We believe that this difference can be explained by differences in the walking ability of the subjects. Many of the subjects in this study were able to independently walk on flat land (FAC score of 4); however, the study by Hermand et al. (2019) included many subjects who needed to be watched while walking in case they began to fall (FAC score of 3). It is likely that the subjects with relatively good walking ability in this study had a high degree of gait automaticity, resulting in unused cognitive resources for the second task. We found a significant negative correlation between PFC activation and gait speed during dual-task interference and no significant difference in the percentage of correct responses to cognitive tasks between single and dual tasks. It has been reported that healthy subjects with good postural control pay more attention to cognitive rather than physical performance during dual-task gait (Mori et al., 2018). The fact that subjects with stable gait in the present study prioritize unused brain resources for cognitive task performance rather than gait speed (similarly to healthy subjects) may be a reason for the significant negative correlation between PFC activation and gait speed during dual-task interference. It is interesting to note that cognitive performance was maintained in the dual task even in the seated stepping task group, indicating that similar results may be obtained in task prioritization by adjusting the difficulty of the motor task, even if there are differences in motor function. These results may help to elucidate the mechanism of task prioritization. The walking task group showed significantly higher O_2_Hb concentrations in the contralesional hemisphere than in the lesional hemisphere, while the seated stepping task group showed a variation in the hemisphere activated by each task. This may be due to differences in cognitive function between the two groups. MMSE was not significantly different between the two groups, but the *p*-value of 0.096 indicates a difference in cognitive function between the two groups, suggesting that the walking task group with higher cognitive function may have been able to use compensatory mechanisms. Cabeza et al. (2002) found that older adults with poorer CT performance used the same PFC regions inefficiently, whereas older adults with better performance used compensatory mechanisms to reorganize the neural network by activating the PFC in both hemispheres. Studies of chronic stroke patients (Liu et al., 2018; Mori et al., 2018) have shown different activity between hemispheres. Hermand et al. (2019) reported that subacute stroke patients showed no differences between hemispheres; furthermore, no differences were found in the seated stepping task group in the present study. Because of the difficulty of performing both walking and CT during the subacute phase, few studies have included subacute stroke patients; therefore, further investigations that include the effects of differences in motor function are necessary.

The frontal pole is the site of measurement within the PFC when using the Pocket NIRS HM. The frontal pole has been reported to be involved in cognitive processes that lead to voluntary actions (Zysset et al., 2002; Haggard, 2008); furthermore, it is preferentially activated when learning new motor tasks (Ishikuro et al., 2014) and has an important role in rehabilitation. It has been suggested that actively increasing PFC activation in older adults may help prevent or delay age-related brain changes (Kawashima et al., 2005; Nishijima et al., 2010), and the PFC has been implicated in executive functions such as attention and working memory, and also in gait through its relationship with the striatum and hippocampus (Pugh and Lipsitz, 2002; Erickson and Barnes, 2003). The results of this study suggest that seated stepping may provide an alternative dual-task condition to walking in patients who have difficulty walking. The results of this study may assist in the rehabilitation of subacute stroke patients with exercises.

This study had several limitations. First, based on the sample size estimation, we set up a seated stepping task group and a walking task group, but not a control group for healthy subjects. Second, the portable NIRS used does not specify Differential Pathlength Factor values, and moreover, the number of channels was only two, located at the BA10 equivalent of the bilateral frontal poles, making it impossible to determine whether the observed activation patterns are PFC-specific. Additional studies are necessary to confirm the involvement of other brain regions. Third, blood pressure and pulse rate variability, which may affect NIRS measurements, were only measured before and after the start of the study to confirm physiological changes. Meanwhile, multiple resolution analysis was performed to minimize physiological noise and motion artifacts in the signal, and deoxygenated hemoglobin concentration was also analyzed to reflect neural activity in the brain more accurately, but the effect of skin blood flow could not be controlled. This is because this would require a multichannel device with separate channels. Fourth, a letter fluency task was used as the second task during this study. Systematic reviews of PFC activation during walking have reported that studies using verbal fluency and subtraction tasks are the most common, with the PFC being activated 83% of the time during the verbal fluency task and 64% during the subtraction task (Pelicioni et al., 2019); however, the pattern of activation of the frontal pole may differ depending on the type of task. Additionally, because the letter fluency task requires the activity of muscles adjacent to the PFC, it cannot be ruled out that muscle activity and facial expressions may have influenced the NIRS signal. Finally, although this study was a cross-sectional study using only a single scale, longitudinal follow-up of the current study may reveal a relationship between dual-tasking ability and neural efficiency. We also believe that future studies looking more closely at the effects of differences in walking ability will elucidate the mechanisms of task prioritization.

This study examined PFC activation during DTSS and DTW performed by subacute stroke patients with hemiplegia and evaluated the association between PFC activation, frontal lobe functions, and dual-task interference. DTSS significantly activated the PFC compared to seated stepping alone, and the hemoglobin concentration during NSS was significantly higher than that during the CT. In the seated stepping task group with low motor function and difficulty walking, the physical demands of the seated stepping task were similar to those of walking. Furthermore, the association between PFC activation, FAB scores, and dual-task interference in the seated stepping task group showed significant positive correlations between PFC activation and cognitive performance with dual-task interference and between FAB scores and cognitive performance with dual-task interference. The higher the frontal lobe function, as indicated by FAB scores, the better the cognitive performance with dual-task interference. Furthermore, when the frontal lobe function is higher, there are more unused brain resources for the dual task, and these unused brain resources may prioritize CTs. The walking task group had significantly higher O_2_Hb concentrations in the contralesional hemisphere than in the ipsilesional hemisphere, possibly because of the availability of compensatory mechanisms. Although many subacute stroke patients are in the process of functional recovery and have difficulty walking, DTSS may be an effective means of activating the PFC of patients who are at low risk for falling and have difficulty walking.

## Data availability statement

The raw data supporting the conclusions of this article will be made available by the authors, without undue reservation.

## Ethics statement

The studies involving human participants were reviewed and approved by Hiroshima University Epidemiological Research Ethics Review Committee (approval no. E-2689-1). The patients/participants provided their written informed consent to participate in this study.

## Author contributions

SN conceived, designed, and performed the experiments and wrote the manuscript. KI performed the experiments and revised the manuscript. KS revised the manuscript. HO conceived and designed the experiments and revised the manuscript. All authors contributed to the article and approved the submitted version.

## Conflict of interest

The authors declare that the research was conducted in the absence of any commercial or financial relationships that could be construed as a potential conflict of interest.

## Publisher’s note

All claims expressed in this article are solely those of the authors and do not necessarily represent those of their affiliated organizations, or those of the publisher, the editors and the reviewers. Any product that may be evaluated in this article, or claim that may be made by its manufacturer, is not guaranteed or endorsed by the publisher.
